# Shifts in Antipsychotic Prescribing by Clinician Type for Medicare Part D Beneficiaries, 2013-2023

**DOI:** 10.1001/jamanetworkopen.2026.3410

**Published:** 2026-03-25

**Authors:** Youngran Kim, Xuan Zhou, Shilei Du, Trudy M. Krause, Rafael Samper-Ternent, Antonio L. Teixeira

**Affiliations:** 1 Department of Management, Policy and Community Health, School of Public Health, The University of Texas Health Science Center at Houston; 2 Center for Health Care Data, School of Public Health, The University of Texas Health Science Center at Houston; 3 Department of Biostatistics and Data Science, The University of Texas Health Science Center at Houston; 4 Institute of Aging, The University of Texas Health Science Center at Houston; 5 The Glenn Biggs Institute for Alzheimer’s & Neurodegenerative Diseases, The University of Texas Health Science Center at San Antonio

## Abstract

**Importance:**

Despite safety warnings, antipsychotics remain commonly prescribed to older adults, particularly those with dementia, and for off-label indications. However, little is known about how prescribing patterns vary by clinician type over time.

**Objective:**

To examine trends in antipsychotic prescribing by clinician type among Medicare beneficiaries from 2013 to 2023.

**Design, Setting, and Participants:**

This repeated cross-sectional study used the Medicare Part D Prescribers by Provider and Drug dataset from 2013 to 2023. Participants included Medicare beneficiaries enrolled in Medicare Part D. Analyses were performed between November 2024 and June 2025.

**Exposure:**

Prescribing clinician type, categorized as psychiatrist, primary care physician (PCP), advanced practice registered nurse (APRN) or physician assistant (PA), or other physician.

**Main Outcomes and Measures:**

Annual antipsychotic prescription claims, the proportion of claims by clinician type, the number of prescribing clinicians, and the mean number of claims per clinician.

**Results:**

From 2013 to 2023, annual antipsychotic prescription claims decreased from 10.8 million to 7.8 million for psychiatrists (average annual percentage change [AAPC], −3.2% [95% CI, –3.7% to –2.7%]; *P* < .001) and from 7.4 million to 5.7 million for PCPs (AAPC, −2.6% [95% CI, –3.2% to –2.3%]; *P* < .001). In contrast, claims prescribed by APRNs or PAs more than tripled from 2013 to 2023, increasing from 3.1 million to 9.5 million (AAPC, 11.8% [95% CI, 10.9%-12.7%]; *P* < .001). The proportion of prescriptions decreased for psychiatrists (48.4% to 32.4%) and PCPs (33.0% to 23.8%) but increased for APRNs and PAs (13.8% to 39.6%) from 2013 to 2023. These shifts were associated primarily with the expanding number of prescribing APRNs and PAs rather than an increase in the mean number of claims per clinician. Although trends were observed in both rural and urban areas, rural areas experienced larger shifts from PCPs to APRNs and PAs, whereas urban areas experienced larger shifts from psychiatrists to APRNs and PAs.

**Conclusions and Relevance:**

In this cross-sectional study of Medicare Part D data from 2013 to 2023, antipsychotic prescribing shifted from psychiatrists and PCPs to APRNs and PAs, reflecting growth in nonphysician prescribers. These findings suggest evolving roles in antipsychotic medication management and highlight the need for appropriate training for the full range of clinician types who prescribe these medications to Medicare enrollees.

## Key Points

**Question:**

How have patterns of antipsychotic prescribing by clinician type changed among Medicare Part D beneficiaries from 2013 to 2023?

**Findings:**

In this repeated cross-sectional study, the proportion of antipsychotic prescriptions written by advanced practice registered nurses and physician assistants increased from 13.8% to 39.6%, making them the largest group of prescribers. During the same period, the proportion of antipsychotic prescriptions written by psychiatrists decreased from 48.4% to 32.4% and by primary care physicians from 33.0% to 23.8%.

**Meaning:**

This study suggests that nonphysician clinicians now account for a larger proportion of antipsychotic prescribing among Medicare beneficiaries, indicating shifts in the mental health care workforce and prescribing practices.

## Teaser

This cross-sectional study examines patterns in antipsychotic prescribing by clinician type, as well as variation by rural vs urban clinician status and across states, among Medicare Part D beneficiaries from 2013 to 2023.

## Introduction

Antipsychotic medications are used to treat a range of behavioral and psychiatric conditions across the lifespan. The US Food and Drug Administration has approved several antipsychotics for schizophrenia and bipolar disorder and as adjunctive therapy for major depressive disorder.^1^ More recently, brexpiprazole was approved for agitation associated with dementia due to Alzheimer disease.^2,3^ Among older adults, antipsychotic use requires caution, as it is associated with increased all-cause mortality.^4^ In particular, among individuals with dementia, antipsychotic use has been associated with a range of serious adverse effects, including sedation, falls, cognitive decline, acute kidney injury, and cardiovascular events.^3,5,6^ Despite their potential for serious adverse effects,^7^ antipsychotics are frequently prescribed off-label to manage behavioral and psychological symptoms among older adults, particularly those with dementia.^8,9^ The 2011 Office of Inspector General report^10^ raised concerns about this practice in nursing homes, prompting federal and state efforts to implement quality monitoring of antipsychotic use in these settings.^11^

Although antipsychotic use has been extensively studied at the patient level, with attention to the prevalence of potentially inappropriate prescribing, safety concerns, and adverse effects,^3,5,6,8,9^ there is comparatively limited understanding of how prescribing patterns vary across clinician types.^12,13^ Previous studies have documented increased antipsychotic prescribing by nonpsychiatrists,^12,14^ but less is known about how the distribution of prescribing across clinician types has changed over time. This knowledge gap is particularly important given the persistent shortage of psychiatrists^15^ and their reduced participation in Medicare^16^ amid increasing demands for mental health services.^17^ The shortage is especially acute in rural and underserved areas, where psychiatrist availability is lowest. Over the past decade, the health care workforce has undergone substantial changes, including the rapid growth of advanced practice registered nurses (APRNs) and physician assistants (PAs) in both mental health and primary care settings.^18^ State and federal policies have increasingly expanded the scope of practice for APRNs. Since the Institute of Medicine endorsed full practice authority for nurse practitioners in 2010, many states have enacted legislation granting APRNs independent authority to diagnose and prescribe medications, although substantial state-level variation remains.^19,20^ At the federal level, Medicare permits APRNs to bill directly for covered services and reimburses these services at 85% of the physician fee schedule, potentially influencing patterns of care delivery.^21,22^ In addition, the availability of lower-cost generic versions of most atypical antipsychotics, after multiple patent expirations,^23,24^ may have reduced financial and administrative barriers^25,26^ and facilitated broader prescribing of antipsychotics by nonpsychiatrists.^27,28^

This study examined national trends in antipsychotic prescribing by clinician type among Medicare Part D beneficiaries from 2013 to 2023. Medicare Part D provides prescription drug coverage for most older adults and many individuals with disabilities, making it a critical source for assessing national prescribing trends.^29,30,31^ We evaluated changes in the proportion of antipsychotic claims by clinician type over time and further assessed variation by rural vs urban clinician status and across states. By characterizing these patterns over a decade, this study provides a descriptive foundation for understanding workforce-related shifts in antipsychotic prescribing across clinician types.

## Methods

### Study Design and Study Participants

We conducted a repeated cross-sectional study using the Centers for Medicare & Medicaid Services (CMS) Medicare Part D Prescribers by Provider and Drug dataset from 2013 to 2023.^32^ The dataset comprises Medicare Part D prescription claims, capturing drugs dispensed to beneficiaries and reporting the total number of filled claims (original prescriptions and refills) aggregated by prescriber National Provider Identifier (NPI), brand name, and generic name. The annual number of beneficiaries enrolled in Medicare and Medicare Part D was obtained from the CMS Medicare Enrollment Report to estimate the population size and served as the denominator for calculating overall prescribing rates and the number of prescribing clinicians per beneficiary across states.^33^ Part D coverage is not mandatory, and approximately 20% to 25% of Medicare beneficiaries have historically opted out.^33^ In 2023, 77.3% of Medicare beneficiaries were enrolled in Part D, including 43.2% in stand-alone Prescription Drug Plans under Medicare fee-for-service and 56.8% in Medicare Advantage Prescription Drug plans.^34^ The analysis was limited to prescribers with state Federal Information Processing Standards codes corresponding to the 50 US states and Washington, DC, excluding overseas territories. The University of Texas Health Science Center at Houston deemed the study exempt from institutional review board review and granted a waiver of informed consent because it was secondary research using publicly available data. The study adhered to the Strengthening the Reporting of Observational Studies in Epidemiology (STROBE) reporting guideline.^35^

### Antipsychotic Drug, Clinician Type, and Rural or Urban Status

We identified typical (first-generation) and atypical (second-generation) antipsychotic drugs flagged as antipsychotics by CMS in the Medicare Part D Specific Drug Lists Report (eTable 1 in Supplement 1).^36^ Clinician type was determined using the Provider Specialty Type description, derived from the Medicare Provider Specialty Code associated with the NPI that had the largest number of Part B claims or, if unavailable, from the primary taxonomy code linked with the NPI.^34^ Clinician types were grouped into 4 categories: psychiatrist, primary care physician (PCP), advanced practice registered nurse (APRN) or physician assistant (PA), and other specialty physician (eTable 2 in Supplement 1).^37^ Nonphysician clinicians other than APRNs or PAs, as defined by their specialty description, were excluded, representing fewer than 0.5% of total antipsychotic claims. Clinicians’ rural or urban status was determined using Rural-Urban Commuting Area codes,^38^ a census tract–based classification system obtained from the Medicare Part D Prescribers by Provider dataset (eTable 3 in Supplement 1).

### Statistical Analysis

The number of antipsychotic prescription claims was aggregated by clinician type for each calendar year. Trends from 2013 to 2023 were assessed using Joinpoint regression models to identify time points with statistically significant changes in trend and to calculate the annual percentage change (APC) for each time segment and the average annual percentage change (AAPC) for the entire study period using Joinpoint regression software, version 5.4.0 (National Cancer Institute).^39^ All statistical tests were 2-sided, and APCs and AAPCs were considered significantly different from zero at α = .05. We calculated the proportion of antipsychotic prescription claims by clinician type, overall and stratified by rural vs urban prescribers. In addition, we reported the number of antipsychotic prescribers by clinician type and the mean annual number of antipsychotic prescription claims per prescriber to assess changes in clinician participation and prescribing intensity over time. For 2023, we assessed the proportion of antipsychotic prescription claims by clinician type across states and generated maps illustrating geographic variation using Microsoft Excel 365 (Microsoft Corp). We also mapped the number of psychiatrists in the Medicare Part D dataset per 100 000 beneficiaries^33^ to explore potential associations between psychiatrist availability and prescribing patterns across states.

## Results

### Trends in Antipsychotic Prescription Claims by Clinician Type

**Figure 1.**
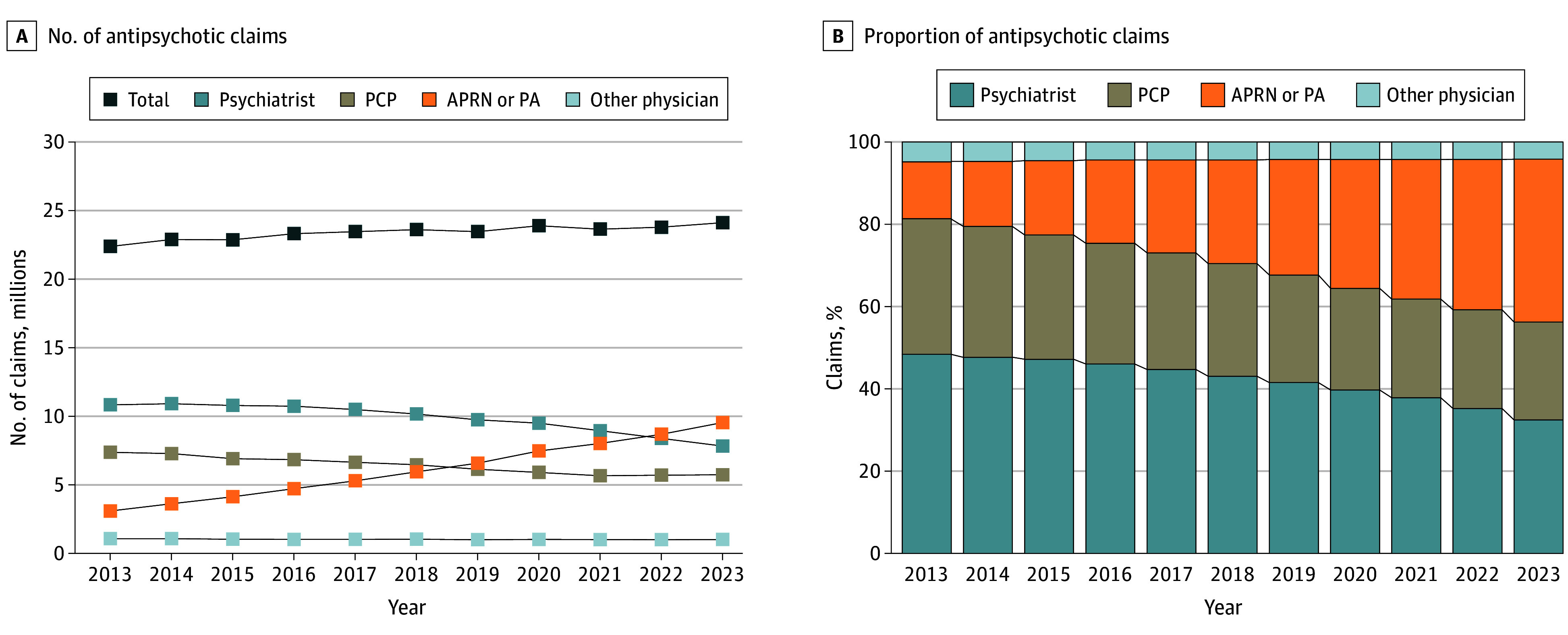
Trends in Antipsychotic Prescription Claims by Clinician Type Among Medicare Part D Beneficiaries, 2013-2023 APRN indicates advanced practice registered nurse; PA, physician assistant; and PCP, primary care physician.

Antipsychotic prescription claims increased from 22.4 million in 2013 to 24.1 million in 2023. During this period, overall Medicare enrollment increased from 52.4 million to 66.5 million individuals. Given that Medicare Part D enrollment increased from 35.7 million to 51.9 million individuals,^33^ antipsychotic claims decreased from 0.63 to 0.46 per enrollee. During this period, the volume and distribution of antipsychotic prescription claims among Medicare Part D beneficiaries shifted markedly across clinician types (Figure 1 and Table). For each clinician type, the Joinpoint model identified at most 1 statistically significant Joinpoint, resulting in 2-time segments per series (Table). No additional significant Joinpoints were detected beyond those reported. Claims by psychiatrists decreased from 10.8 million to 7.8 million (Figure 1), reflecting an AAPC of –3.2% (95% CI, –3.7% to –2.7%; *P* < .001) (Table). The most pronounced decrease occurred between 2018 and 2023, with an APC of –5.2% (95% CI, –6.7% to –4.2%; *P* < .001). Similarly, claims by PCPs decreased from 7.4 million to 5.7 million (Figure 1), with an AAPC of –2.6% (95% CI, –3.2% to –2.3%; *P* < .001) (Table). In contrast, claims prescribed by APRNs or PAs more than tripled, increasing from 3.1 million in 2013 to 9.5 million in 2023 (Figure 1). This reflects an AAPC of 11.8% (95% CI, 10.9%-12.7%; *P* < .001), with a steep APC of 14.0% per year (95% CI, 12.7%-17.9%; *P* < .001) from 2013 to 2018, followed by continued growth from 2018 to 2023 with an APC of 9.6% (95% CI, 6.4%-11.0%; *P* < .001) (Table). Claims by other physician specialties remained relatively stable, ranging from 1.0 to 1.1 million annually (Figure 1), with an AAPC of –0.7% (95% CI, –1.0% to –0.4%; *P* < .001) (Table). As a result of these shifts, the proportion of claims prescribed by APRNs and PAs increased markedly from 13.8% in 2013 to 39.6% in 2023, surpassing all other clinician types by the end of the study period (Figure 1). Over the same period, the share of claims from psychiatrists decreased from 48.4% to 32.4%, and the share of claims from PCPs decreased from 33.0% to 23.8%.

**Table.** Annual Changes in Antipsychotic Prescription Claims by Clinician Type and Rural or Urban Status Among Medicare Part D Beneficiaries, 2013-2023^a^

Clinician type and period	APC for the segmented period (95% CI), %	*P* value	AAPC for 2013-2023 (95% CI), %	*P* value
**All**				
Overall				
2013-2016	1.3 (0.3 to 3.3)	.005	0.7 (0.3 to 1.0)	<.001
2016-2023	0.4 (−1.4 to 1.0)	.21		
Psychiatrist				
2013-2018	−1.1 (−2.1 to 0.7)	.11	−3.2 (−3.7 to −2.7)	<.001
2018-2023	−5.2 (−6.7 to −4.2)	<.001		
PCP				
2013-2021	−3.3 (−5.3 to −1.4)	.01	−2.6 (−3.2 to −2.3)	<.001
2021-2023	−0.2 (−3.2 to 1.7)	.56		
APRN or PA				
2013-2018	14.0 (12.7 to 17.9)	<.001	11.8 (10.9 to 12.7)	<.001
2018-2023	9.6 (6.4 to 11.0)	<.001		
Other physician				
2013-2016	−1.9 (−3.7 to −0.6)	.008	−0.7 (−1.0 to −0.4)	<.001
2016-2023	−0.2 (−0.7 to 1.4)	.72		
**Rural**				
Overall				
2013-2021	−0.4 (−2.1 to 1.6)	.14	0.1 (−0.4 to 0.4)	.84
2021-2023	1.9 (−0.5 to 3.5)	.16		
Psychiatrist				
2013-2017	−1.7 (−2.6 to −0.8)	.01	−4.4 (−4.7 to −4.2)	<.001
2017-2023	−6.2 (−6.7 to −5.7)	<.001		
PCP				
2013-2021	−4.1 (−6.4 to −1.4)	.005	−3.4 (−4.1 to −3.0)	<.001
2021-2023	−0.9 (−4.2 to 1.2)	.24		
APRN or PA				
2013-2015	13.6 (11.2 to 15.7)	<.001	10.2 (9.8 to 10.5)	<.001
2015-2023	9.3 (8.7 to 9.6)	<.001		
Other physician				
2013-2023	−5.0 (−6.3 to −3.6)	<.001	−5.0 (−6.3 to −3.6)	<.001
**Urban**				
Overall				
2013-2017	1.4 (0.9 to 2.6)	<.001	0.8 (0.6 to 1.0)	<.001
2017-2023	0.4 (−0.5 to 0.7)	.14		
Psychiatrist				
2013-2018	−1.0 (−2.0 to 1.1)	.17	−3.0 (−3.6 to −2.5)	<.001
2018-2023	−5.1 (−6.8 to −4.0)	<.001		
PCP				
2013-2021	−3.0 (−4.6 to −2.5)	<.001	−2.4 (−2.9 to −2.1)	<.001
2021-2023	0 (−2.7 to 1.6)	.77		
APRN or PA				
2013-2018	14.8 (13.5 to 17.4)	<.001	12.3 (11.6 to 13.0)	<.001
2018-2023	9.8 (7.4 to 11.1)	<.001		
Other physician				
2013-2016	−1.4 (−2.8 to −0.4)	<.001	−0.2 (−0.4 to 0.1)	.21
2016-2023	0.4 (0.1 to 1.1)	.009		

Abbreviations: AAPC, average annual percentage change; APC, annual percentage change; APRN, advanced practice registered nurse; PA, physician assistant; PCP, primary care physician.

^a^ Trends were quantified using Joinpoint regression software, version 5.4.0 (National Cancer Institute). The optimal number of Joinpoints was selected using the grid search method to detect changes in trends. The APC was estimated for segmented periods, and the AAPC was estimated for the years 2013 to 2023.

### Trends in Antipsychotic Prescription Claims by Clinician Type and Rural or Urban Status

**Figure 2.**
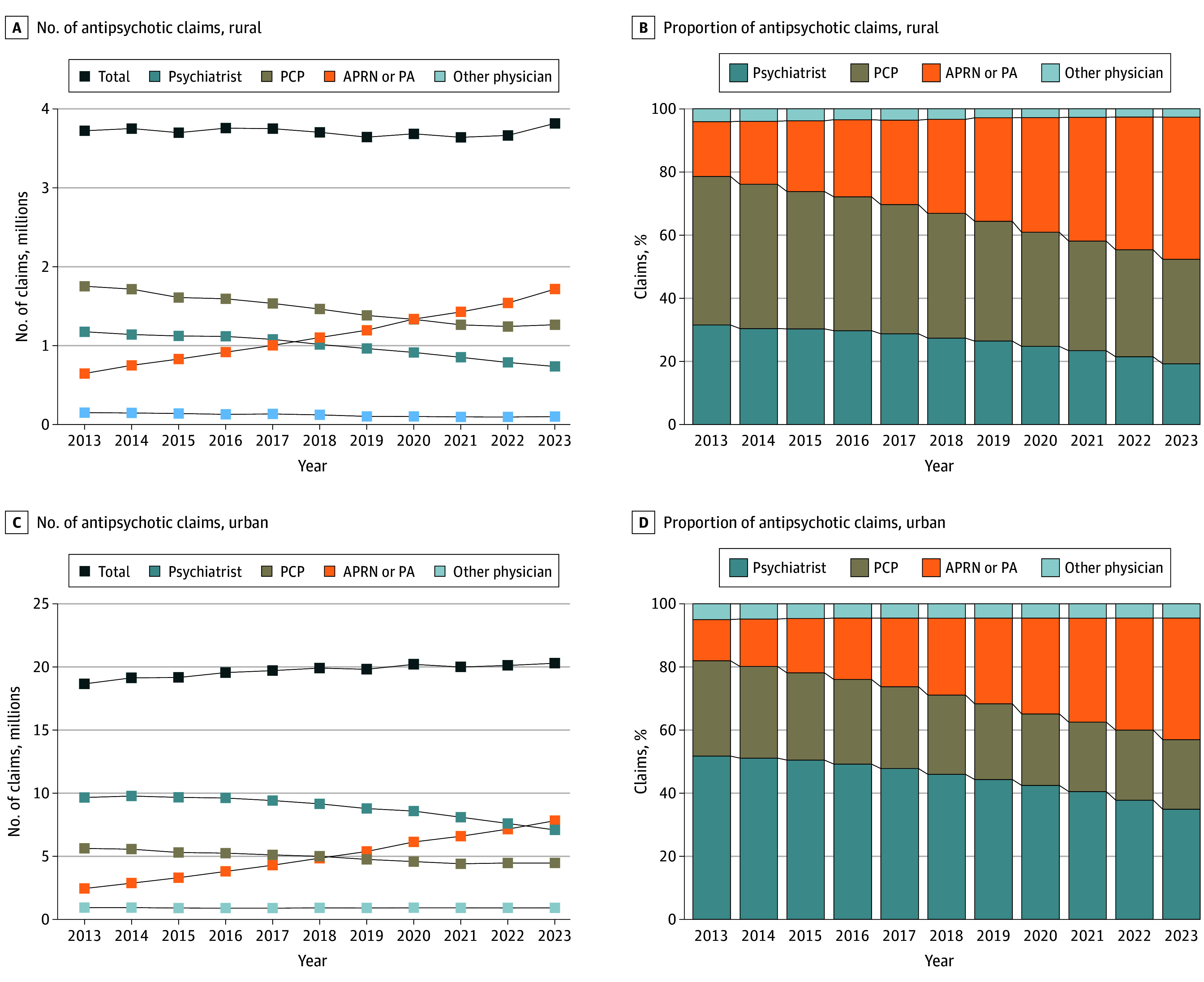
Trends in Antipsychotic Prescription Claims by Clinician Type and Rural or Urban Status Among Medicare Part D Beneficiaries, 2013-2023 APRN indicates advanced practice registered nurse; PA, physician assistant; and PCP, primary care physician.

When stratified by clinician rural or urban status, similar shifts were observed across clinician types in both settings (Table and Figure 2). In rural areas, PCPs accounted for the largest share of antipsychotic claims in 2013 (47.1%) (Figure 2), but their volume steadily decreased over the decade (AAPC, –3.4% [95% CI, –4.1% to –3.0%]; *P* < .001) (Table). During the same period, APRN and PA claims in rural areas nearly tripled, increasing from 17.3% of claims in 2013 to 45.0% in 2023, surpassing all other clinician types (Figure 2). In urban areas, psychiatrists accounted for more than half of antipsychotic claims in 2013 (51.8%), but their share decreased to 34.9% by 2023. Meanwhile, APRN and PA claims in urban areas increased substantially, from 2.4 million in 2013 to 7.8 million in 2023 (Figure 2), reflecting an AAPC of 12.3% (95% CI, 11.6%-13.0%; *P* < .001) (Table). Correspondingly, the proportion of claims from APRNs and PAs in urban areas increased from 13.1% in 2013 to 38.5% in 2023 (Figure 2).

### Trends in Number of Prescribing Clinicians and Mean Claims Per Clinician

**Figure 3.**
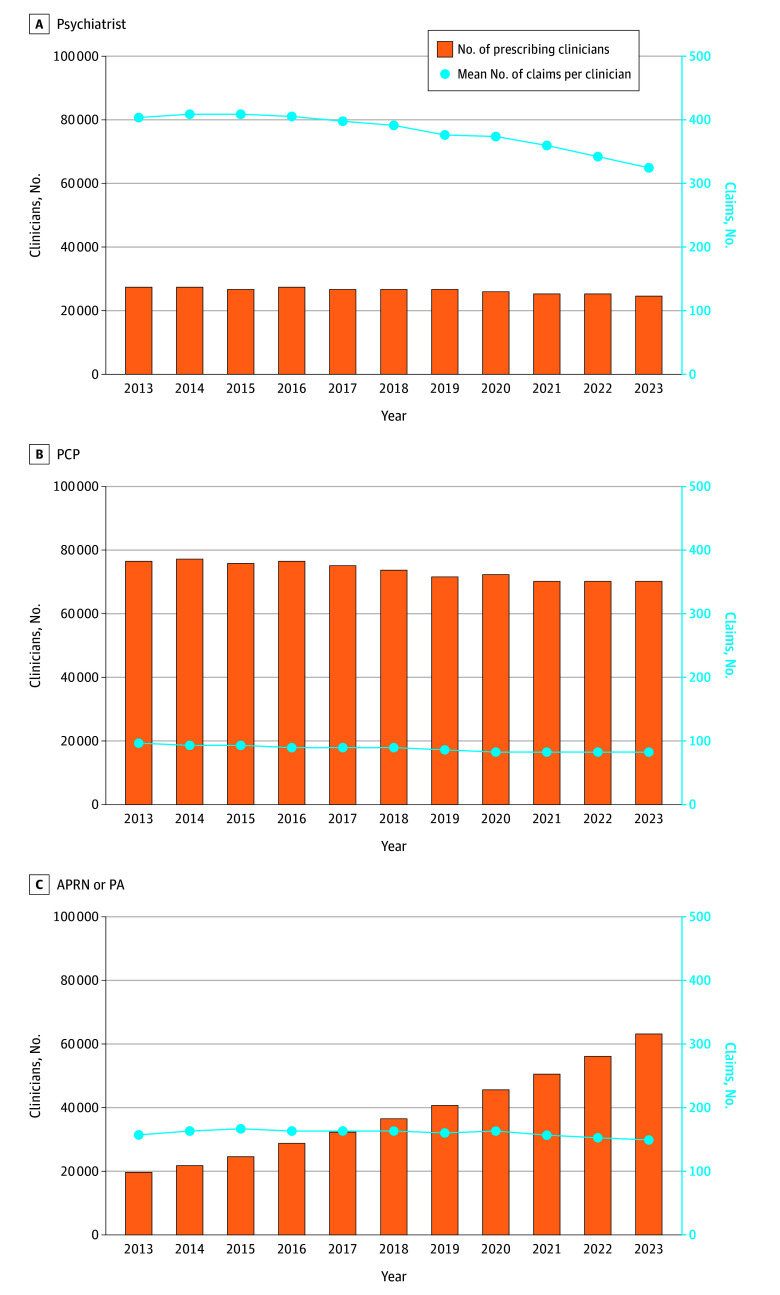
Trends in the Number of Antipsychotic Prescribers and Average Claims Per Prescriber by Clinician Type Among Medicare Part D Beneficiaries, 2013-2023 APRN indicates advanced practice registered nurse; PA, physician assistant; and PCP, primary care physician.

From 2013 to 2023, both the number of Medicare Part D clinicians prescribing antipsychotics and the mean annual number of antipsychotic claims per clinician varied substantially by clinician type (Figure 3). Among psychiatrists, the number of prescribing clinicians decreased from 27 219 in 2013 to 24 953 in 2023, while the mean number of claims per psychiatrist decreased from 407.5 to 326.3. For PCPs, the number of prescribers decreased from 76 893 to 70 498, and their mean number of claims per clinician decreased from 97.0 to 83.0. In contrast, the number of APRNs and PAs prescribing antipsychotics more than tripled, increasing from 19 554 in 2013 to 63 154 in 2023. Their mean number of claims per clinician remained relatively stable, ranging from 158.3 to 151.1 during the study period.

### Proportion of Antipsychotic Prescription Claims by Clinician Type Across States

**Figure 4.**
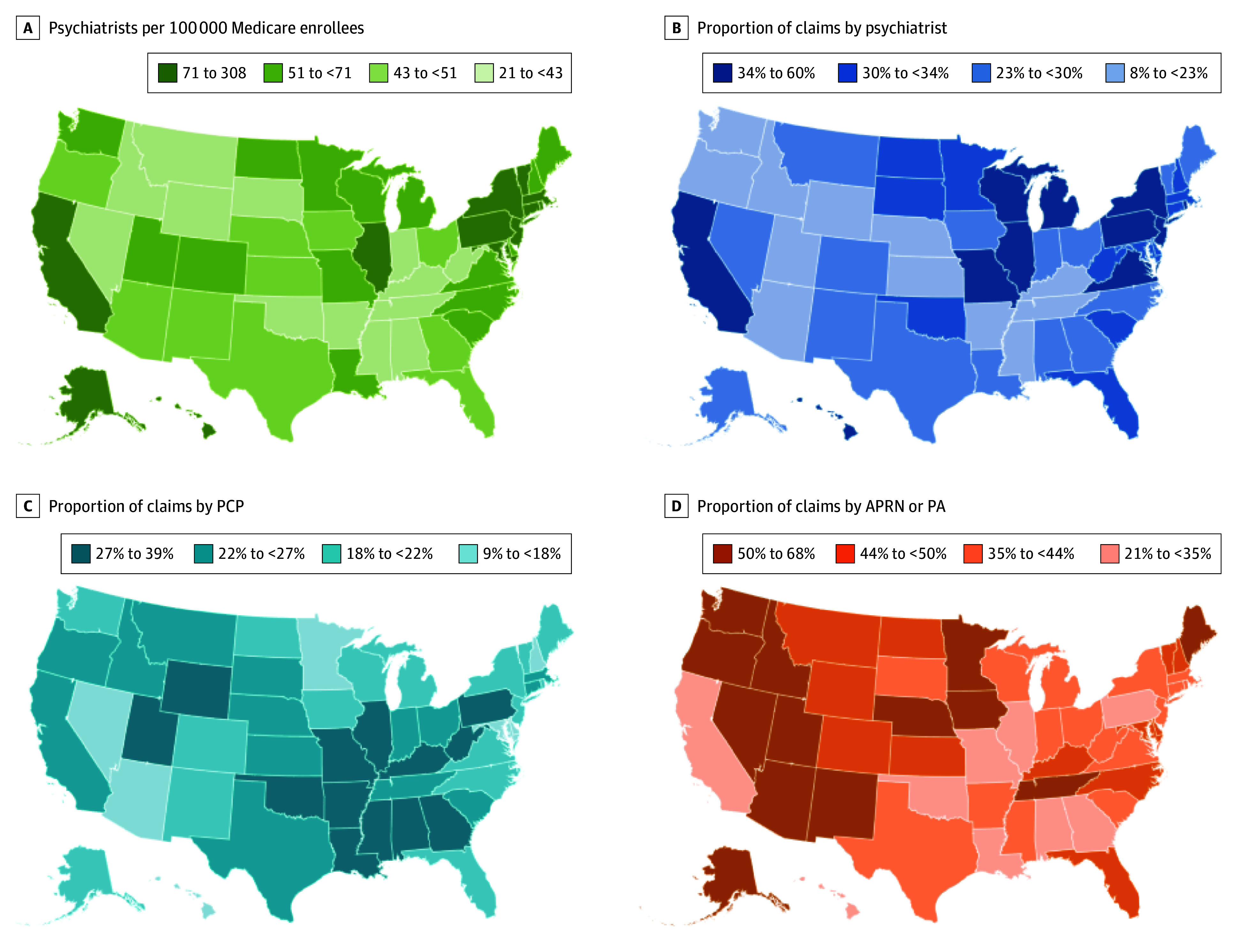
Map Showing Proportion of Antipsychotic Prescription Claims by Clinician Type Across States Among Medicare Part D Beneficiaries, 2023 APRN indicates advanced practice registered nurse; PA, physician assistant; and PCP, primary care physician.

Figure 4 shows geographic variation in psychiatrist availability and the proportion of antipsychotic prescription claims by clinician type. Psychiatrist availability, measured as the number of psychiatrists per 100 000 Medicare beneficiaries, varied widely across states (Figure 4A). States with greater psychiatrist availability generally had higher proportions of psychiatrist-prescribed claims, particularly in California and in several states in the northeast and midwest (Figure 4B). In contrast, in states with lower psychiatrist availability, southern states tended to have higher proportions of claims from PCPs (Figure 4C), whereas western and midwestern states tended to have higher proportions of claims from APRNs and PAs (Figure 4D).

## Discussion

In this national analysis of Medicare Part D data from 2013 to 2023, we observed substantial shifts in antipsychotic prescribing across clinician types. The share of prescriptions from psychiatrists and PCPs decreased, while prescriptions from APRNs and PAs increased markedly. By 2023, APRNs and PAs accounted for nearly 40% of all antipsychotic claims, surpassing psychiatrists and PCPs. These trends were evident in both rural and urban areas, although APRNs and PAs reached higher relative shares in rural areas. Geographic variation further showed that states with fewer psychiatrists per Medicare beneficiary tended to have higher proportions of prescribing by PCPs or APRNs and PAs.

We observed that total antipsychotic prescription claims increased from 22.4 million in 2013 to 24.1 million in 2023; however, this increase likely reflects growth in the Medicare population rather than higher prescribing rates. During this period, overall Medicare enrollment increased from 52.4 million to 66.5 million individuals with the US population aging, and Medicare Part D enrollment increased from 35.7 million to 51.9 million individuals.^33^ Accordingly, prescriptions per beneficiary enrolled in the pharmacy benefit actually decreased, consistent with prior reports.^1,8,9,23^ After the 2011 Office of Inspector General report that identified widespread off-label use of atypical antipsychotics in nursing homes,^10^ CMS began publicly reporting antipsychotic use among nursing home residents without psychotic conditions as a quality measure for nursing homes,^11^ and antipsychotic prescribing among nursing facility residents has decreased substantially.^8,9^

Our study demonstrates a clear shift in antipsychotic prescribing from psychiatrists and PCPs to APRNs and PAs, consistent with prior studies showing the expanding role of APRNs and PAs in both overall medication prescribing and behavioral health prescribing.^40,41^ Over the study period, the number of psychiatrists and PCPs prescribing antipsychotics decreased, while the number of APRNs and PAs more than tripled in our analysis. The mean number of prescriptions per APRN or PA remained relatively stable, suggesting that growth in prescribing was associated with more nonphysician clinicians entering the prescribing pool rather than existing prescribers increasing their volume. These findings highlight workforce changes in mental health and the expanding role of nonphysician clinicians in managing patients receiving antipsychotics, particularly in areas with limited psychiatrist availability.^18,42^ National reports have consistently documented a critical shortage of psychiatrists, indicating that the psychiatric workforce is insufficient to meet current needs, with projections suggesting substantial shortfalls in the coming decades. The Health Resources and Services Administration has estimated that demand for psychiatrists will continue to outpace supply, particularly in rural and underserved areas.^43^ Shortages are further compounded by the aging of the psychiatrist workforce, with a large proportion nearing retirement age, and limited numbers of trainees entering the field.^44^ Moreover, psychiatrists have the highest Medicare opt-out rate among all specialties, at 8.1% in 2024, representing nearly 40% of all physicians who have opted out,^45^ and have provided fewer services to Medicare beneficiaries over time,^16^ which may further exacerbate access challenges. This combination of workforce contraction and Medicare nonparticipation may have shifted much of the responsibility for prescribing and managing psychotropic medications to other clinician groups. Among those groups, PCPs were early primary prescribers of antipsychotics in rural areas but have also decreased in number. This decrease likely reflects ongoing integration of mental health care into primary care, where screening, diagnosis, and treatment of psychiatric conditions are increasingly handled in primary care settings,^15,46,47,48,49^ as well as broader workforce shortages in primary care specialties that are projected to worsen over time.

In contrast, APRNs and PAs have increasingly taken on these prescribing roles. Psychiatric mental health nurse practitioners, who have specialized training and certification, may help offset workforce shortages and play a growing role in managing psychiatric needs among older adults.^49,50,51^ Family nurse practitioners, who represent the largest share of the nurse practitioner workforce, may also be prescribing antipsychotics in primary care settings, particularly in rural areas,^49^ where they may be replacing PCPs. Prior studies have shown rapid growth of nurse practitioners in nursing homes,^51^ where off-label antipsychotic use is more common for managing behavioral and psychological symptoms of dementia. Although the present study was not stratified by care setting, the increase in APRN and PA prescribing may reflect their expanding role in nursing homes rather than a uniform shift across all Medicare Part D enrollees. Future research should distinguish APRNs and PAs by specialty to clarify their respective contributions to antipsychotic prescribing, as well as examine whether these trends vary by practice model (independent vs team based), prescribing setting (community vs nursing home), mode of care delivery (in person vs telehealth), and prescription type (new vs refill). Understanding these dynamics will be critical for designing policies that ensure access to mental health care while safeguarding appropriate and evidence-based prescribing.

Although our state-level analysis was limited, patterns observed highlight notable variation. As expected, in states with greater psychiatrist availability, psychiatrists account for more antipsychotic prescriptions, whereas in states with fewer psychiatrists, PCPs or APRNs often play a more prominent role. These findings suggest that prescribing regulations, scope-of-practice laws, and other requirements for nonphysician clinicians may be associated with these differences.^50^ In states with lower psychiatrist availability, southern states tended to have higher proportions of claims by PCPs, whereas states in the west and midwest regions with fewer psychiatrists generally had higher proportions of antipsychotic claims by APRNs and PAs. According to the Scope of Practice Policy database, states allowing broader scope of practice, including independent practice without physician supervision and expanded prescribing authority for APRNs and PAs, are concentrated in the west and midwest.^52,53^ These policy differences may be associated with the observed patterns of larger proportions of antipsychotic prescriptions by APRNs and PAs in these states.^52,53^ Further investigation into these policy factors could clarify the sources of variation and inform workforce planning.

### Limitations

This study has several limitations. First, Medicare Part D data do not capture prescriptions paid for out of pocket or through other insurance, potentially underestimating total prescribing volumes. Second, we used aggregated clinician-level data for each drug, which did not include patient-level demographic or clinical characteristics, residential status, or the specific clinical indication for antipsychotic use. As a result, we could not examine differences in prescribing patterns by patient factors such as age, sex, race and ethnicity, comorbidities, diagnoses, care setting, or whether antipsychotics were prescribed for approved vs off-label indications. Third, prescriber specialty is based on self-reported codes and may be subject to misclassification. Also, APRNs and PAs were grouped together as a single nonphysician category; however, PAs accounted for a much smaller share of prescribing than APRNs, suggesting that the observed trends are likely associated primarily with APRNs. Furthermore, specialty information was not available for nonphysician clinicians, making it impossible to isolate psychiatric mental health nurse practitioners from other APRNs. Fourth, while we examined psychiatrist availability by state, other factors such as local prescribing norms, scope-of-practice regulations, and telehealth use may be associated with observed patterns but were not measured.

## Conclusions

In this cross-sectional study of Medicare Part D data from 2013 to 2023, the distribution of antipsychotic drugs prescribed among Medicare Part D beneficiaries shifted from psychiatrists and PCPs to APRNs and PAs, who accounted for the largest share of prescriptions by the end of the study period. These findings highlight substantial changes in clinician-level antipsychotic prescribing patterns across the US, particularly in areas with limited psychiatrist availability.

## Notes

Supplement 1. **eTable 1.** List of Antipsychotic Drugs **eTable 2.** Definitions of Clinician Type **eTable 3.** Categorization of Rural-Urban Commuting Area Codes (RUCAs)

Supplement 2.
Data Sharing Statement
