# Supplementary material for: Shifts in Antipsychotic Prescribing by Clinician Type for Medicare Part D Beneficiaries, 2013-2023
Source: JAMA Netw Open. 2026 Mar 25;9(3):e263410. doi: 10.1001/jamanetworkopen.2026.3410 (PMC13019238; doi:10.1001/jamanetworkopen.2026.3410)
Supplement: Supplement 1. — eTable 1. List of Antipsychotic Drugs eTable 2. Definitions of Clinician Type eTable 3. Categorization of Rural-Urban Commuting Area Codes (RUCAs) [file jamanetwopen-e263410-s001.pdf]

Supplementary Online Content

Kim Y, Zhou X, Du S, Krause TM, Samper-Ternent R, Teixeira AL. Shifts in antipsychotic prescribing by clinician type for Medicare Part D beneficiaries, 2013-2023. *JAMA Netw Open.* 2026;9(3):e263410. doi:10.1001/jamanetworkopen.2026.3410

eTable 1. List of Antipsychotic Drugs

eTable 2. Definitions of Clinician Type

eTable 3. Categorization of Rural-Urban Commuting Area Codes (RUCAs)

This supplementary material has been provided by the authors to give readers additional information about their work.

© 2026 Kim Y et al. *JAMA Network Open.*

eTable 1. List of Antipsychotic Drugs

Typical	Atypical
Chlorpromazine	Aripiprazole
Fluphenazine	Asenapine
Haloperidol	Brexpiprazole
Loxapine	Cariprazine
Perphenazine	Clozapine
Pimozide	Iloperidone
Thioridazine	Lumateperone
Thiothixene	Lurasidone
Trifluoperazine	Olanzapine
	Paliperidone
	Pimavanserin
	Quetiapine
	Risperidone
	Ziprasidone

© 2026 Kim Y et al. *JAMA Network Open.*

eTable 2. Definitions of Clinician Type

**Clinician Type**	**Provider Specialty Type Description**
Psychiatrist	Geriatric Psychiatry, Neuropsychiatry, Psychiatry & Neurology, Psychiatry
Primary Care Physician	Obstetrics & Gynecology, Family Medicine, Family Practice, Pediatric Medicine, General Practice, Internal Medicine
Advanced Practice Registered Nurse/ Physician Assistant	Nurse Practitioner, CRNA, Certified Registered Nurse Anesthetist (CRNA), Certified Nurse Midwife, Registered Nurse,* Certified Clinical Nurse Specialist, Physician Assistant
Other Specialty Physician	Colon & Rectal Surgery, Radiology, Micrographic Dermatologic Surgery, Integrative Medicine, Oral & Maxillofacial Surgery, Peripheral Vascular Disease, Clinical Cardiatric Electrophysiology, Independent Medical Examiner, Hematopoietic Cell Transplantation and Cellular Therapy, Clinical Cardiac Electrophysiology, Phlebology, Hand Surgery, Colorectal Surgery (formerly proctology), Surgical Oncology, Neurological Surgery, Plastic Surgery, Advanced Heart Failure and Transplant Cardiology, Thoracic Surgery (Cardiothoracic Vascular Surgery), Gynecological/Oncology, Cardiac Electrophysiology, Interventional Radiology, Cardiac Surgery, Surgery, Maxillofacial Surgery, Pain Medicine, Colorectal Surgery (Proctology), General Acute Care Hospital, Radiation Oncology, Neuromusculoskeletal Medicine, Sports Medicine, Vascular Surgery, Sports Medicine, Undefined Physician type, Interventional Cardiology, Hematology, Gynecological Oncology, Hospital, Allergy/Immunology, Orthopaedic Surgery, Plastic and Reconstructive Surgery, Nuclear Medicine, Hospital (Dmercs Only), Neurosurgery, Ophthalmology, Allergy/ Immunology, Legal Medicine, Thoracic Surgery, Urology, Hematology/Oncology, Otolaryngology, Dermatology, Orthopedic Surgery, Cardiovascular Disease (Cardiology), Sleep Medicine, Critical Care (Intensivists), Preventive Medicine, Interventional Pain Management, Diagnostic Radiology, Medical Oncology, Pain Management, Anesthesiology, Endocrinology, Rheumatology, Gastroenterology, Osteopathic Manipulative Medicine, Addiction Medicine, Hematology-Oncology, Infectious Disease, Hospice and Palliative Care, Specialist, General Surgery, Nephrology, Cardiology, Pulmonary Disease, Physical Medicine and Rehabilitation, Hospitalist, Emergency Medicine, Geriatric Medicine, Neurology, Unknown Physician Specialty Code.

*We assumed that registered nurses in the Medicare Part D Prescribers–by Provider and Drug dataset were advanced practice registered nurses, as their credentials indicated an advanced degree.

© 2026 Kim Y et al. *JAMA Network Open.*

eTable 3. Categorization of Rural-Urban Commuting Area Codes (RUCAs)

Categorization	RUCAs
Rural	4.0, 4.2, 5.0, 5.2, 6.0, 6.1, 7.0, 7.2, 7.3, 7.4, 8.0, 8.2, 8.3, 8.4, 9.0, 9.1, 9.2, 10.0, 10.2, 10.3, 10.4, 10.5, and 10.6
Urban	1.0, 1.1, 2.0, 2.1, 3.0, 4.1, 5.1, 7.1, 8.1, and 10.1

© 2026 Kim Y et al. *JAMA Network Open.*
