# Supplementary material for: Shifts in Antipsychotic Prescribing by Clinician Type for Medicare Part D Beneficiaries, 2013-2023
Source: JAMA Netw Open. 2026 Mar 25;9(3):e263410. doi: 10.1001/jamanetworkopen.2026.3410 (PMC13019238; doi:10.1001/jamanetworkopen.2026.3410)
Supplement: Supplement 2. — Data Sharing Statement [file jamanetwopen-e263410-s002.pdf]

Data Sharing Statement

Kim. Shifts in Antipsychotic Prescribing by Clinician Type for Medicare Part D Beneficiaries, 2013-2023. *JAMA Netw Open*. Published March 25, 2026.
doi:10.1001/jamanetworkopen.2026.3410

Data
Data available: Yes
Data types: Data (not involving human participants), Data dictionary
How to access data: The data is publicly available through the CMS website:
https://data.cms.gov/provider-summary-by-type-of-service/medicare-part-d-prescribers/medicare-part-d-prescribers-by-provider-and-drug
When available: With publication

Supporting Documents
Document types: None

Additional Information
Who can access the data: The data is publicly available through the CMS website.
Types of analyses: The data is publicly available through the CMS website.
Mechanisms of data availability: The data is publicly available through the CMS website:
https://data.cms.gov/provider-summary-by-type-of-service/medicare-part-d-prescribers/medicare-part-d-prescribers-by-provider-and-drug
